# Necroptosis-related lncRNAs: establishment of a gene module and distinction between the cold and hot tumors in glioma

**DOI:** 10.3389/fonc.2023.1087117

**Published:** 2023-04-21

**Authors:** Kangxi Cao, Fengbo Su, Xuchun Shan, Xingyu Jiang, Zhaohui Ni, Yan Chen

**Affiliations:** ^1^Department of Neurosurgery, The Second Hospital of Jilin University, Changchun, China; ^2^Department of Pathogenobiology, The Key Laboratory of Zoonosis, Chinese Ministry of Education, College of Basic Medical Sciences, Jilin University, Changchun, China

**Keywords:** necroptosis, glioma, lncRNA, cold and hot tumors, prognosis

## Abstract

**Background:**

Gliomas are the most common primary tumors of the central nervous system and portend a poor prognosis. The efficacy of emerging and promising immunotherapies varies significantly among individuals. Distinction and transformation of cold and hot tumors may improve the antitumor efficacy of immunotherapy.

**Methods and Results:**

In this study, we constructed a necroptosis-related lncRNA module based on public databases. The association of this module with survival was assessed using the Cox regression, Kaplan-Meier survival analysis, and nomogram, external validation was also conducted in another public database. Furthermore, we performed gene set enrichment analysis (GSEA), immune checkpoint and tumor microenvironment analysis, and *in vitro* qRT-PCR validation. Finally, we clustered all samples into 2 clusters based on the expression of model lncRNAs and identified cluster 1 as cold tumors with fewer infiltrating T cells.

**Conclusions:**

Identifying cold and hot tumors by necroptosis-related lncRNAs can help available immunotherapeutic strategies to achieve efficacy in the precise treatment of individuals. Prior treatment failure can be overcome by targeting necroptosis-related lncRNAs.

## 1 Introduction

Gliomas are the most common primary brain tumors, accounting for about 80% of central nervous system malignancies (1). The World Health Organization (WHO) classified gliomas into grades 1, 2, 3, and 4. Grades 1 and 2 are low-grade gliomas (LGGs), whereas grades 3 and 4 are high-grade gliomas (HGGs) (2). Survival outcomes in patients with HGG are generally poor. The median overall survival (OS) of patients with grade 3 gliomas is three years, while the OS time of patients with grade 4 gliomas, especially glioblastoma (GBM), is only about 15 months, and less than 5% of patients survive five years after diagnosis (3, 4). Currently, the primary treatments for gliomas are mainly surgery resection, radiotherapy, and chemotherapy (5). These treatments, however, are not always practical. Electrotherapy has showed some potential in the treatment of GBM in recent years. Due to a lack of understanding of its underlying mechanisms and controversial clinical trial results, however, it is still not wildly accepted by the medical community (6). Immunotherapy is a burgeoning treatment approach that has been well-studied in various preclinical and clinical studies with dramatic responses (1), but treatment failures and side effects are frequently reported (7). Using immune checkpoint (ICP) inhibitors is a kind of immunotherapy; however, only one-third of patients respond to them (8). This is mostly due to a lack or poor infiltration of tumor T cells, which leads to resistance to ICP inhibitors and results in “cold tumors,” which are characterized by a lack of pre-existing immune cell infiltration (9). To exploit immunotherapy’s full potential and overcome this obstacle, it is crucial to explore ways to differentiate between cold and hot cancers and transform cold tumors into hot tumors.

Necroptosis is a type of programmed cell death that characterized by swollen organelles, ruptured plasma membranes, and the release of damage-associated molecular pattern (DAMP) elements to disseminate secondary inflammation (10). Necroptotic signaling is tightly intertwined with many regulatory pathways and contributes to fundamental physiological responses such as inflammation, immune responses, embryonic development, and maintenance of tissue homeostasis. Moreover, the precise regulation of the major necroptotic mediators RIPK1, RIPK3, and MLKL is crucial for maintaining a delicate balance of TME between cell survival and necroptotic death (10). Ectopic introduction of necrotic cells into the tumor microenvironment (TME) activates BATF3+/cDC1 as well as CD8^+^ leukocytes to trigger anti-tumor immune responses. In contrast, tumor-associated antigen-presenting cells increase the tumor antigen load (11). The treatment of targeting necroptosis mechanisms is increasingly recognized as a promising therapeutic strategy (8). Osthole could induce necroptosis of glioma cells *via* ROS generation and so may have therapeutic potential for glioma treatment (12). Bufalin was reported to kill glioma cells by inducing apoptosis or necroptosis (13). Melo-Lima et al. found that edelfosine could trigger rapid and massive glioma cells necroptosis through the promoter of RIP1/RIP3 necrosomes *in vitro* (14). These data imply that necroptosis induction may represent a novel strategy for glioma immunotherapy. However, necroptosis-associated molecular markers in glioma have not been adequately reported.

Long non-coding RNA (lncRNAs) are endogenous non coding RNAs with length of more than 200 nucleotides. They have been proved to have crucial roles in glioma angiogenesis, tumor growth, infiltration, and metastasis through epigenetic level, transcription and post transcriptional modification (15). Some lncRNAs can interact with microRNAs, thus preventing miRNAs from interacting with their target mRNA. For example, lncRNA CASC2 could inhibit malignancy in human gliomas *via* miR-21 (16). LncRNA H19 affects glioma angiogenesis and the biological activity of glioma-associated endothelial cells by suppressing microRNA-29a (17). Due to their abnormal expression in glioma patients’ brain tissue, cerebrospinal fluid, or peripheral circulation, some lncRNAs are thought to be potential biomarkers. Just recently, some necroptosis-related lncRNAs have also been identified which could be used to assess the prognosis and molecular characteristics of glioma (18–20). However, it is unknown if these lncRNAs are implicated in distinct glioma immune microenvironments or if they may be exploited as novel markers to distinguish cool from hot tumors and as prospective immunotherapy targets for glioma.

To address the aforementioned issue, this study conducted a series of analyses to discover the hidden wonders of necroptosis-related lncRNAs and glioma features of cold and hot, with the goal of discovering a completely new way to improve glioma prognosis.

## 2 Materials and methods

### 2.1 Transcriptome and clinical data of glioma

We downloaded transcriptomic and clinical data of 698 glioma patients and five normal controls from the Cancer Genome Atlas (TCGA) database (https://portal.gdc.cancer.gov/). Then, we used the software Perl (version 5.32) to transfer the ensemble id to the gene id. To reduce statistical bias, we excluded glioma patients with missing information on overall survival (OS) or with short OS (< 30 days). Finally, we obtained 627 samples with clinical information and randomly divided them into training and test groups in a 1:1 ratio.

### 2.2 Acquisition of necroptosis-related genes and lncRNAs

We conducted a literature review and obtained 67 necroptosis-related genes (NRGs) and 14,057 related lncRNAs. Necroptosis-related lncRNAs were identified by Pearson correlation analysis (coefficients > 0.4, p< 0.001). Next, we obtained 190 differentially expressed necroptosis-related lncRNAs (|log_2_ fold change [FC]| > 2, false discovery rate [FDR]< 0.01) by R package ‘limma’.

### 2.3 Construction and validation of risk module

After the identification of differential expression lncRNAs, normal samples were excluded in the following analyses. Then, we performed a univariate cox (uni-cox) regression analysis to screen out necroptosis-related lncRNA associated with survival (p< 0.05). We then performed Lasso regression with 10-fold cross-validation and a p-value of 0.05 for 1,000 loops. To avoid overfitting, random stimulation was set 1,000 times for each cycle. Next, multiple cox regression (multi-cox) was conducted, and the signature below was used to calculate the risk score:


$$RiskScore=\sum_{k=1}^{n}coef\left(lncRNA^{k}\right)\;*\;expr\left(lncRNA^{k}\right)$$

where the *coef (lncRNA)* was the coefficient of lncRNAs obtained in multi-cox and *expr (lncRNA)* was the expression of lncRNAs. Then, we plotted the module's 1-, 3-, and 5-year time-dependent receiver operating characteristics (ROC) curves. We divided samples into low-risk and high-risk groups according to the median value of the risk score.

### 2.4 Identification of independent factors and ROC curves

We applied univariate Cox (uni-Cox) and multivariate Cox (multi-Cox) regressions to evaluate the prognostic, predictive power of the risk score and clinical characteristics, and plotted ROC curves to compare the predictive outcomes of different factors. Furthermore, we constructed a nomogram based on the risk score, age, gender, and tumor grade using the R package ‘rms’. Moreover, calibration curves based on the Hosmer-Lemeshow test were plotted to judge whether the prediction showed good agreement with the practice.

### 2.5 External database validation

The glioma lncRNA sequencing and clinical data were downloaded from the CGGA database (http://cgga.org.cn/index.jsp) and acquired 173 samples in total. Furthermore, using the formula proposed and calculating the risk scores of each sample, then all samples were grouped into high-risk and low-risk groups. In the next stage, the expression and survival differences of the 9 lncRNAs in the two groups were compared to justify the accuracy of the formula. The value p 0.05 denoted statistical significance.

### 2.6 Cell culture

NHA, U87, U251, and A172 cell lines were grown under 5% CO_2_ at 37°C in Dulbecco’s modified eagle medium (DMEM; Gibco, NY, USA) supplemented with 10% fetal bovine serum (FBS) and 1% penicillin-streptomycin. All cell lines in this study were obtained from the Shanghai Cell Bank of the Chinese Academy of Medical Sciences (Shanghai, China).

### 2.7 qRT-PCR analysis

After total RNA was extracted with a Total RNA Extraction kit (Solarbo, Beijing, China), reverse transcription was conducted using a first-strand cDNA synthesis kit (Invitrogen, Carlsbad, CA, USA) following the manufacturer’s protocols. RT-PCR was then performed using the Premix Ex Taq SYBR Green PCR kit (TaKaRa, Dalian, China), following the manufacturer’s instructions. The primers used in this experiment are summarized in **Supplement Table 1**.

### 2.8 Investigation of immune infiltration and tumor microenvironment

To explore the correlation between the risk score and immune infiltration, we used the EPIC algorithm to calculate the immune infiltration status among the glioma patients from the TCGA by R packages ‘limma’, ‘scales’, ‘ggplot2’, and ‘ggtext’. Besides, we compared TME scores between low- and high-risk groups by R package ‘ggpubr’.

### 2.9 Gene set enrichment analysis

Based on a curated gene set (Kegg.v7.5.symbols.gmt), we applied GSEA software (version 4.01) (http://www.gsea-msigdb.org/gsea/index.jsp) to identify the significantly enriched pathways between risk groups. p< 0.05.

### 2.10 Unsupervised clusters based on nine prognostic lncRNAs

To explore the response to immunotherapy in patients with glioma, we identified potential molecular subgroups by R package ‘ConsensusClusterPlus’ based on the expressions of prognostic lncRNAs. Principal component analysis (PCA), t-distributed stochastic neighbor embedding (t-SNE), and Kaplan-Meier survival were subsequently performed using the R package ‘Rtsne’. Besides, we made immunity analysis and immune checkpoint analysis.

### 2.11 Exploring the correlation between the 9 lncRNAs and pharmaceutical therapy response

To further illustrate the application potential of the proposed necroptosis-related signature, we extracted 421 samples that were treated by pharmaceuticals from the 627 samples obtained from the TCGA database. Then, these samples were grouped into two groups: the long-term survival group and the short-term survival group based on the median of the 421 samples’ survival times. Next, using the Wilcoxon rank-sum test, the expression of the 9 lncRNAs and the risk scores were compared between the long-term survival and the short-term survival groups, and p 0.05 was considered statistically significant.

### 2.12 Statistical analysis

We mainly used R software to perform data visualization and statistical analyses. For survival analysis, the samples were divided into high- and low-risk groups based on the median value of the risk score. The survival difference was determined by the KM method. Wilcoxon rank-sum test was used for the expression difference of necroptosis-related lncRNAs. We used Pearson’s correlation analysis to determine the correlations of necroptosis-related genes and necroptosis-related lncRNAs, and uni-Cox analysis to profile survival-related lncRNAs. The qRT-PCR results were presented as the mean ± standard error of the mean (SEM) and screened by ANOVA. In all the statistical tests, p (or FDR)< 0.05 was considered statistically significant.

## 3 Results

### 3.1 Necroptosis-related lncRNAs in glioma

**Figure 1:**
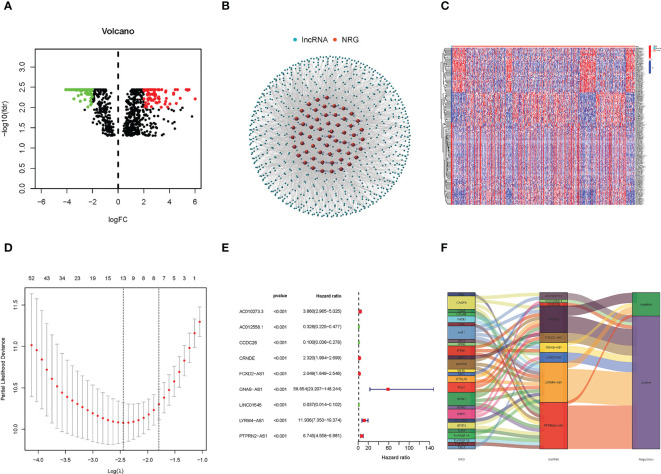
Identification of Necroptosis-related lncRNAs and construction of the prognostic model of necroptosis-related lncRNAs. **(A)** The heatmap of differentially expressed lncRNAs. The red points on the right of the vertical axis represent up-regulated in the tumor samples compared with normal samples. On the contrary, the green points on the lift of the vertical axis represent down-regulated. **(B)** The correlation of NRG and necroptosis-related lncRNAs. Red represents NRG, and blue represents necroptosis-related lncRNAs. **(C)** The expression heatmap of significantly differential expressed necroptosis-related lncRNAs, and up- and down-regulated lncRNAs were only exhibited 50 respectively. **(D)** The LASSO coefficient profile of 9 necroptosis-related lncRNAs. **(E)** The forest plot of the lncRNAs extracted to construct the prognostic model. **(F)** The Sankey plot of necroptosis genes and related lncRNAs.

We acquired five normal and 698 glioma patients from the TCGA database. Based on the expression of NRG and differentially expressed necroptosis-related lncRNAs (|log_2_ fold change (FC)| > 2, false discovery rate (FDR)< 0.01) between normal and tumor samples, we finally obtained 190 differentially expressed lncRNAs in which 100 were down-regulated, and 90 were upregulated (**Figure 1A**). The correlations between NRG and necroptosis-related lncRNAs are shown in **Figure 1B**.

### 3.2 Construction and verification of the prognostic model

A total of 166 necroptosis-related lncRNAs were identified to be significantly correlated with the survival of glioma patients based on the uni-Cox regression analysis (p< 0.05), and its expression heatmap was shown in **Figure 1C**. Then, based on the Lasso regression, we finally acquired nine lncRNAs related to necroptosis in glioma when the first-rank value of log(λ) was the minimum likelihood of deviance (**Figure 1D**). In addition, the forest plot of the nine lncRNAs was drawn and shown in **Figure 1E**, and in the nine lncRNAs, AC012558.1, CCDC26, and LINC01545 were low-hazard lncRNAs, because their hazard ratios were lower than 1. And the correlation network of NRGs and necroptosis-related lncRNAs was exhibited in the Sankey diagram (**Figure 1F**). Finally, the risk score was calculated using the following formula for every sample:

Risk score = *PTPRN2-AS1* * (0.8303) + *CCDC26* * (-1.1168) + *FOXD2-AS1* * (0.2859) + *CRNDE* * (0.3229) + *LYRM4-AS1* * (0.7113) + *AC012558.1* * (-0.7539) + *GNAS-AS1* * (1.6708) + *LINC01545* * (-1.9787) + *AC010273.3* * (0.6858).

**Figure 2:**
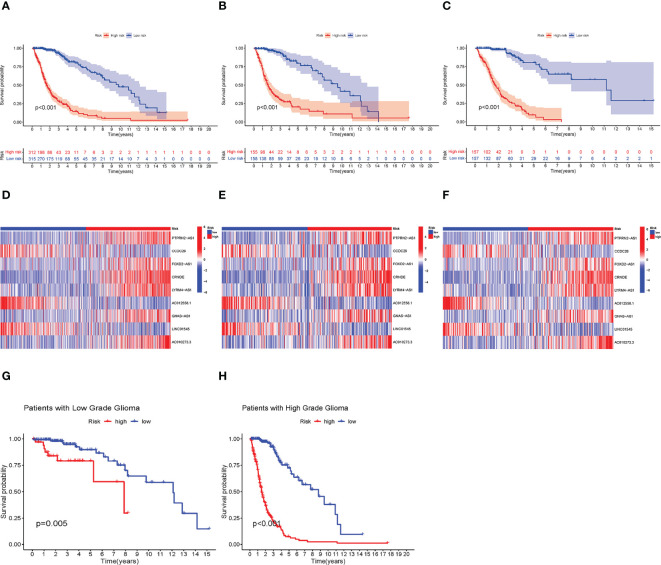
Verification of the prognostic model. **(A–C)** Kaplan–Meier (KM) survival curves of entire, test, and train groups, respectively. **(D–F)** The expression difference of 9 necroptosis-related lncRNAs between low- and high-risk groups in the entire test, and train group, respectively. **(G, H)** The KM survival curves of low- and high-grade Glioma, respectively. Construction of Nomogram and Assessment of the Prognosis Model.

We found that high-risk groups had a poorer prognosis than the low-risk group in both test and training groups (**Figures 2A–C**) (A, B, and C represent the entire, test and training groups, respectively). The expression heatmap of the nine lncRNAs (**Figures 2D–F**) showed that AC012558.1, CCDC26, and LINC01545 were expressed highly in low-risk groups (D, E, and F represent the entire, test, and train groups, respectively). Furthermore, we discovered that the high-risk group had a worse prognosis regardless of whether they had high- or low-grade gliomas (**Figures 2G, H**).

**Figure 3:**
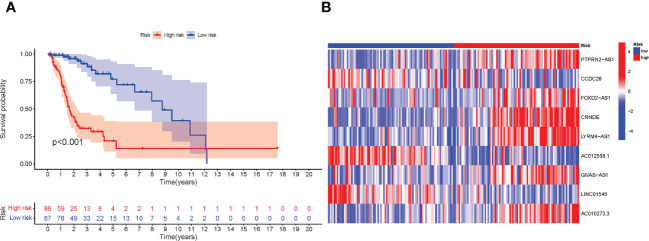
External validation in CGGA. **(A)** Kaplan–Meier (KM) survival curves of all CGGA samples. **(B)** The expression difference of nine necroptosis-related lncRNAs between low- and high-risk groups in CGGA samples.

To validate the accuracy of the module, external validation was conducted on the CGGA database. As shown in **Figures 3A, B**, the expression of the nine lncRNAs showed a similar trend to that of TCGA, and the low-risk group has a better prognosis.

### 3.3 Construction of nomogram and assessment of the prognosis model

**Figure 4:**
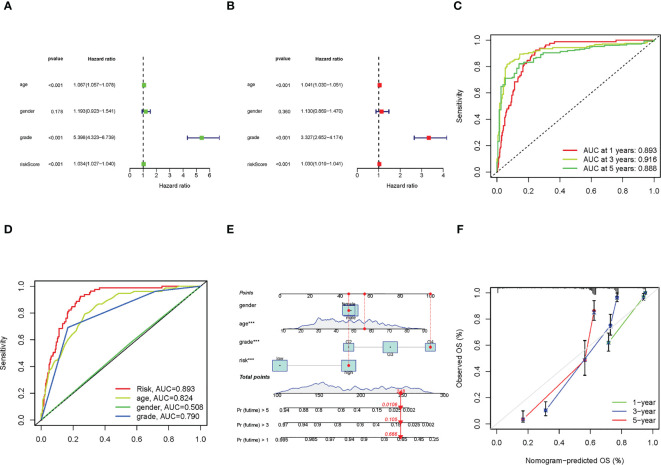
The construction of nomogram and assessment of the prognosis model. **(A, B)** The results of uni-Cox and multi-Cox analysis, respectively. **(C)** The risk score’s ROC curves of 1-, 3-, 5-year OS. **(D)** The ROC curves of risk score, age, gender, and tumor grade, respectively. **(E)** The nomogram that integrated the risk score, age, gender, and tumor grade predicted the probability of the 1-, 3-, and 5-year OS. **(F)** The calibration curves for 1-, 3-, and 5-year OS, respectively.

We used uni-Cox and multi-Cox analyses to confirm the risk score as an independent prognostic factor of clinical features (**Figures 4A, B**). In uni-Cox analysis, the hazard ratio (HR) for the risk score was 1.034, and the 95% confidence interval (CI) was 1.027-1.040 (p< 0.001), and in multi-Cox analysis, the HR was 1.030, and the 95% CI was 1.019-1.041 (p< 0.001). These results identified the risk score as an independent prognostic factor. Furthermore, age and glioma grade were also independent prognostic factors in glioma patients.

To quantify the accuracy of the prognostic module and independent factors, we plotted the time-dependent receiver operating characteristic (ROC) to assess the sensitivity and specificity of the model for prognosis, and we also calculated the area under the ROC curve (AUC). The 1-, 3-, and 5-year AUCs of risk scores were 0.893, 0.916, and 0.888, respectively (**Figure 4C**), and the AUCs of risk scores, age, gender, and grade were 0.893, 0.824, 0.508 and 0.790, respectively (**Figure 4D**). Based on the above results, we established a nomogram predicting the 1-, 3-, and 5-year OS in glioma patients (**Figure 4E**). Furthermore, corresponding calibration plots were generated to demonstrate the nomogram’s robust and stable predictive power (**Figure 4F**), the adjusted lines were around the diagonal, which means the nomogram was accurate.

### 3.4 *In vitro* qRT-PCR validation of bioinformatic results

**Figure 5:**
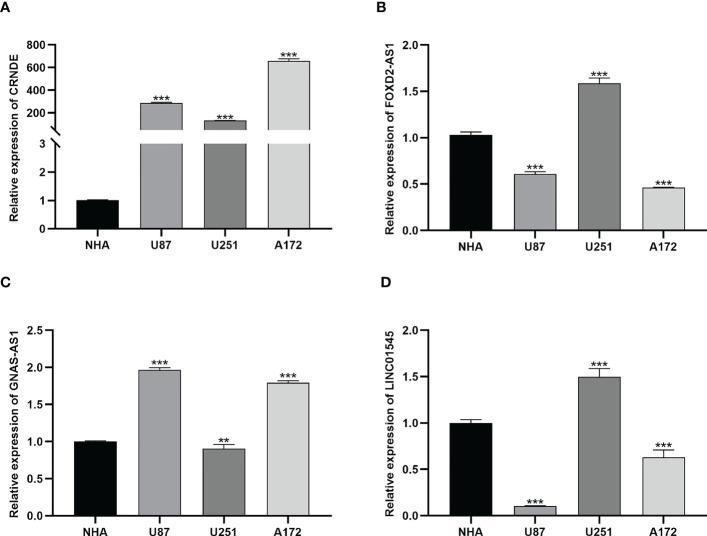
The results of qRT-PCR. **(A)** The expression of CRNDE in NHA, U87, U251 and A172 cell lines. **, and *** represent p< 0.05, p< 0.01, and p< 0.001, respectively, statistically analyzed by ANOVA. **(B–D)** The expression of FOXD2-AS1, GNAS-AS1 and LINC01545 in NHA, U87, U251 and A172 cell lines, respectively.

To validate the bioinformatics results, we utilized qRT-PCR to detect the mRNA expressions of specific lncRNAs, including *CRNDE*, *FOXD2-AS1*, *GNAS-AS1*, and *LINC01545* (**Figure 5**). *CRNDE* was overexpressed in all glioma cell lines (**Figure 5A**), and *FOXD2-AS1* was overexpressed in U251 cells (**Figure 5B**). The long noncoding RNA *GNAS-AS1* associated with cancer metastasis was overexpressed in U87 and A172 cells but at low levels in U251 cells (**Figure 5C**). Moreover, *LINC01545* was upregulated in U251 cells but not in U87 and A172 cells (**Figure 5D**).

### 3.5 Immune infiltration, TME, and GSEA in risk groups

**Figure 6:**
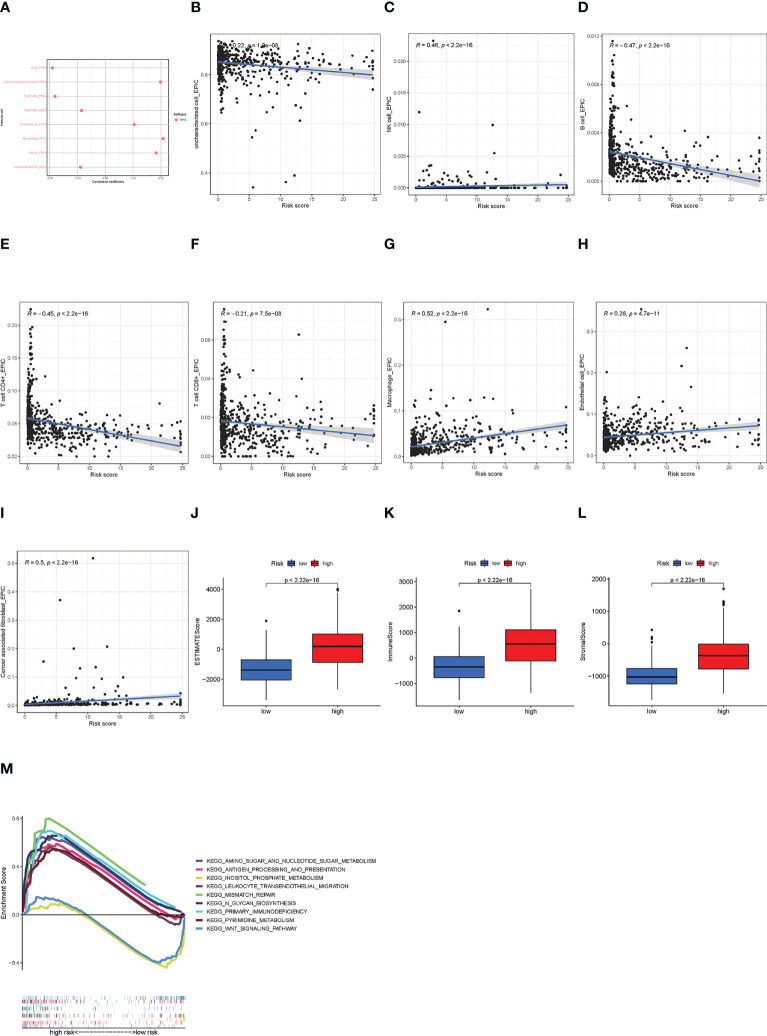
The immune analysis of high- and low-risk groups. **(A)** The immune cell bubble of risk groups. **(B–I)** The correlation of immune cell and risk scores at EPIC. **(J–L)** The TME scores between risk groups. **(M)** GSEA of the eight pathways significantly enriched in the high-risk group and two enriched in the low-risk group.

The correlations between infiltrated immune cells and risk scores are shown in **Figure 6A**. We could find that B cells, CD4^+^ T cells, and CD8^+^ T cells were negatively related, while macrophages and NK cells were positively related to the risk score. The specific correlations between these cells and risk scores are shown in **Figures 6B–I**. In addition, we conducted a TME analysis and got three TME scores, as shown in **Figures 6J–L**. In the three TME scores, the immune score is related to immune cells and we could find that there exists a significant difference between the low- and the high-risk groups (high group > 0, low group< 0) (**Figure 6K**). These results indicated that tumors in the high-risk group had more infiltrated immune cells, signifying a different immune TME from the low-risk group. Then we explored the KEGG pathway in all samples and found biological functions that were significantly different between low- and high-risk groups. Finally, we obtained a total of 10 pathways, eight of which were enriched in the high-risk group and the others in the low-risk group (NOM *p*< 0.05) (**Figure 6M**). Notably, the KEGG pathways enriched in the high-risk group were primarily immune-related. Therefore, we next attempted to perform an immunity analysis in the module.

### 3.6 Distinguishment of the cold and hot tumors

**Figure 7:**
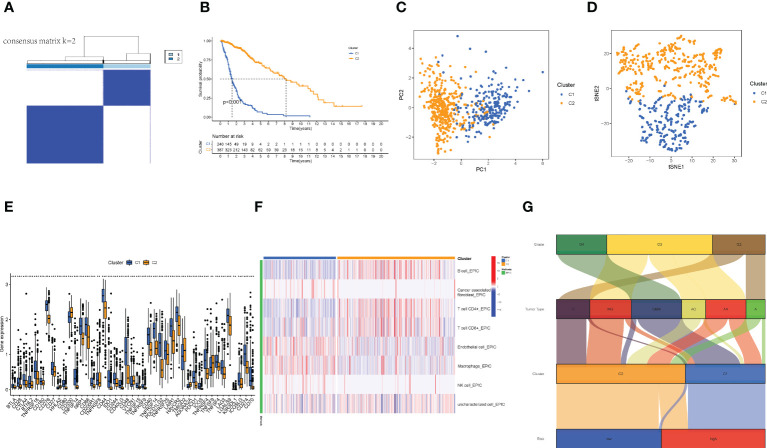
The distinguish between Cold and Hot tumors. **(A)** Patients divided into two clusters by Consensus Cluster Plus. **(B)** The survival of different clusters (p< 0.001). **(C, D)** The PCA and t-SNE of cluster 1 and 2, respectively. **(E)** The difference of 43 checkpoints expression in clusters. **(F)** The immune infiltration difference between clusters. **(G)** The Sankey plot of grade, tumor type, clusters, and risk groups.

Referring to previous studies (21, 22), different clusters (termed as subtypes or different risk groups) usually exhibit distinct immune microenvironments, leading to different immunotherapeutic responses in patients. Therefore, according to the different expression values of the nine lncRNAs, by using Consensus Clustering method, we regrouped the glioma patients into clusters 1 and 2 (**Figure 7A**). Patients in the two clusters had significantly different survival outcomes (**Figure 7B**). Specifically, patients in cluster 1 had poorer survival than that in cluster 2 (*p*< 0.001). The PCA and t-SNE results together also showed satisfactory clustering (**Figures 7C, D**). Furthermore, genes encoding immune checkpoints showed higher expressions in cluster 1 samples (*p*< 0.05) (**Figure 7E**). Then, immune infiltration analysis showed more T cells and B cells infiltration in cluster 2 samples, while cluster 1 samples had more macrophage infiltration but almost no NK cell infiltration (**Figure 7F**). These cells were reported to affect the integrity of the blood-brain barrier (BBB) and promote tumor angiogenesis (23), suggesting that the gliomas in cluster 1 were cold tumors and generally had a poor prognosis.

Finally, we made a Sankey plot (**Figure 7G**). From the plot, we could find that cluster 1 almost corresponded to the high-risk group, and GBM almost corresponded to both cluster 1 and the high-risk group. These results may explain the unfavorable outcomes of GBM patients. In addition, the distinction between cold and hot tumors offered a new approach to precision therapy for glioma patients.

### 3.7 The necroptosis-related signature is positively associated with pharmaceutical therapy response

**Figure 8:**
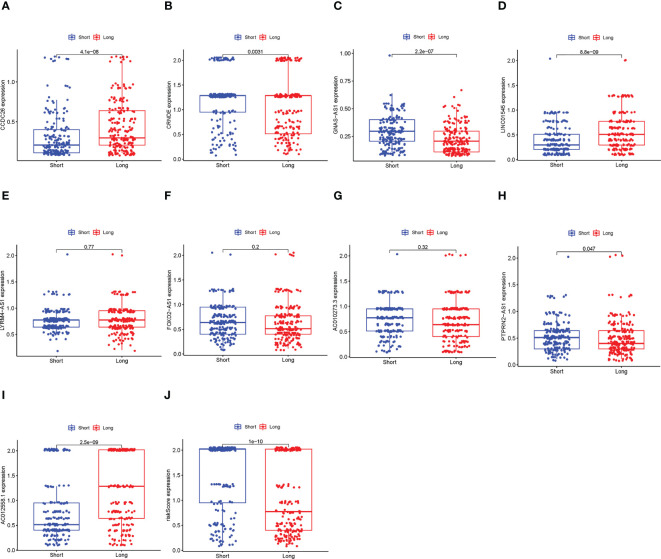
Comparison between the long-term survival and short-term survival groups. **(A–I)** The differential expression alnlysis between the long-term survival and the short-term survival groups of the 9 lncRNAs, p< 0.05 was considered statically sianificant. **(J)** The differential expression alnlysis between the long-term survival and the short-term survival groups of risk scores.

The results of this part are shown in **Figure 8**. Among the 9 lncRNAs, six of them were expressed significantly differently between the long-term survival and short-term survival groups, as well as the risk scores, indicating that the proposed signature and related lncRNAs also had the potential to predict the effect of pharmaceutical therapy.

## 4 Discussion

Primary glioma treatment comprises surgical resection, radiotherapy, chemotherapy, and newly proposed electrotherapy (1, 6). However, they do not consistently work. The frequent failure of glioma treatment due to a cold TME, namely low infiltration of T cells, imposes an enormous burden on the economy and adversely affects patients’ quality of life. Therefore, more methods are needed and immunotherapy may be the last resort. Necroptosis, a novel kind of cell death, is essential to the development of the tumor immune microenvironment and has been proven that could promote the progression of glioma. Moreover, it has been discovered that lncRNAs function as competitive RNAs to affect the genes involved in necroptosis. Therefore, the purpose of this study is to develop a module based on necroptosis-related lncRNAs to predict the prognosis and immunological profile of patients with glioma (24), in addition, to emerge the hidden mechanism of turning cold glioma into hot.

We retrieved glioma clinical and transcriptome data from the TCGA database and constructed a risk signature based on the differentially expressed necroptosis-related lncRNAs that are associated with prognosis. The module consists of nine lncRNAs, and most of the lncRNAs identified in the module have been previously described in the context of glioma, and some of them are related to star genes such as *IDH1*, which was reported to be significantly associated with the prognosis of patients with GBM. Previous research indicates that CCDC26 inhibits the growth and migration of glioma cells *via* targeting miR-203 (25). FOXD2-AS1 acts as a sponge for miR-185-5p and promotes tumorigenesis and the progression of glioma by regulating the HMGA2/PI3K/Akt pathway (26). CRNDE could promote the malignant progression of glioma by attenuating the miR-384/PIWIL4/STAT3 axis (27). GNAS-AS1 has been reported to be related to the malignant progression of osteosarcoma, breast cancer, nasopharyngeal carcinoma, and non-small cell lung cancer (28–31), however, its function in glioma remains unclear. In this study, we determined that GNAS-AS1 overexpression may contribute to poor prognosis in glioma patients. Similarly, this is the first time LYRM4-AS1, which has been linked to osteoarthritis and cervical cancer (11, 15), has been documented in the field of glioma. Among the nine lncRNAs we obtained, three were expressed higher in the low-risk group. In particular, CCDC26, which we considered a low-risk lncRNA for glioma survival and expressed higher in the low-risk group, has been identified as having the function of inhibiting the growth and migration of glioma cells. Most of the roles of the necroptosis-related lncRNAs we found in this study match what was known about their roles in gliomas and other tumors. Prior to this, little research has investigated the functions of PTPRN2-AS1, AC012558.1, LINC01545, and AC0110273.3 in terms of their potential roles in tumors, and research about them is still needed.

Based on the established module of necroptosis-related lncRNAs, Kaplan–Meier analysis, univariate Cox regression, multivariate Cox regression, and nomogram were employed to validate and evaluate the module. The results demonstrated that the module could be used as a sensitive indicator for predicting the 1-, 3-, and 5-year OS in glioma patients, as well as distinguishing cold and hot gliomas. We then verified the expression of four lncRNAs in three glioma cell lines, U87, U251, and A172, by performing qRT-PCR experiments. CRNDE was overexpressed in all glioma cell lines. However, the expression levels of three lncRNAs, FOXD2-AS1, GNAS-AS1, and LINC01545, differed between the three glioma cell lines. This anomalous behavior may be explained by the following factors: Firstly, patients are categorized according to risk scores, which are determined using the signature suggested in the method section. As a result, some of the nine lncRNAs may express less in abnormal cells than expected. Secondly, the three glioma cell lines that we employed in our work may be linked to various patients, and their outcomes varied from those of the samples in the TCGA database, so the expression of the lncRNAs also differed. Thirdly, the lncRNAs’ differential expression may have a crucial role in glioma development and progression. The precise expression levels of all necroptosis-related lncRNAs should be confirmed in biological specimens in the future. This is also a limitation of this study.

Immunotherapy can improve the situation of frequently reported treatment failure; it, however, is not a panacea for all tumors (8). Therefore, we proposed an immune-based classification of cold and hot tumors to improve immunotherapy. While risk groups based on the median risk score could predict prognosis, nonetheless, we could not identify hot and cold tumors. Previous research has reported that molecular subtypes, also known as clusters, are associated with tumor immunosuppression in microenvironments (32, 33). Different clusters confer different immune and TME scores, leading to different prognoses and immunotherapy responses (22, 34). In this study, we grouped patients into two clusters by using the Consensus Clustering method based on the expression values nine lncRNAs listed above and attempted to distinguish between cold and hot tumors (35). There were fewer T cells, higher immune scores, and higher expressions of *IDO1*, *LAG3*, and *CTLA4* in cluster 1, which could be identified as cold tumors. A previous study showed elevated CTLA4 expression in glioma patients correlated with cancer progression (1). Furthermore, emerging evidence indicated that IDO activation was involved in cancer development by assisting tumor cells in evading immune surveillance (36). A recent systematic review and meta-analysis revealed that high expression of IDO1 was associated with poor prognosis in patients with various types of cancer including gliomas (37). Higher immune checkpoint gene expression may be associated with a bad prognosis since it can alter the TME in various ways. CTLA4, for example, could impair T-cell priming by interfering with the DC-T cell contact (38).

The unique TME in the central nervous system contributes to the specific immune landscape of gliomas (39, 40). A variety of peripheral immune cells exist in the glioma microenvironment, including myeloid-derived suppressor cells (MDSCs), natural killer (NK) cells, macrophages, neutrophils, CD4^+^ helper T (Th) cells, CD8^+^ cytotoxic T lymphocytes (CTLs), and regulatory T cells (Treg) (41, 42). However, their infiltration ratio in gliomas is low compared to other solid tumors. In our results, 89% of GBMs belonged to cluster 1 with a poor prognosis, which is consistent with the conclusion of a previous study (43). Immune infiltration analysis indicated that samples in cluster 1 had a lower abundance of T cells but a higher abundance of macrophages, the latter being associated with poor prognosis in patients with gliomas. Macrophages play essential roles in neoplasia, metastasis, immune escape, and angiogenesis (44–49). Therefore, the poor survival of patients with cold tumors may be associated with insufficient immune cell infiltration. Improving T-cell infiltration could tansform cold tumors into hot tumors, although it is not easy. Several methods have been identified in the past for achieving this objective, including driving T-cells into tumors, promoting T-cell priming, boosting the number of antigen-specific T-cells, and promoting T-cell trafficking and infiltration (43). These are merely theoretical methods, and additional research is requiered, despite the fact that some preliminary attempts have yielded promising results. Currently, two primary methods of adoptive T cell immunotherapies are being utilized: first, chimeric antibody receptor engineered T cells (CAR-T), which focus on assisting immune cells in recognizing tumor antigens and then directing them to locate and kill the target tumor cells. Antigen-specific T cells are also utilized to transfect the tumor-specific genetically modified T cell receptors (TCR) and assist in removing tumor immune tolerance (50). The FDA has officially approved the use of CAR-Ts that target the CD19 antigen for the treatment of refractory acute lymphoblastic leukemia/lymphoma, mantle cell lymphoma, and large B cell lymphomas (51–53). However, there are some obstacles to its application in solid tumors. Another study on the use of TCR in the treatment of sarcoma yielded excellent results, demonstrating that boosting infiltrating T cells and transforming a cold tumor into a hot one have therapeutic potential in the treatment of cancer. In solid tumors, for instance, tailored neoantigen vaccines have demonstrated promise in multiplying and diversifying tumor-specific T lymphocytes. Immune-hot melanoma patients (NCT01970385) and immune-cold glioblastoma patients (NCT01970385) have exhibited long-lasting therapeutic improvement from these techniques (54–56). Our signature accurately identifies cool gliomas, which have lower proportions of T-cell infiltration and larger proportions of macrophage infiltration, as well as higher expression of immune checkpoint genes such as CTLA4 and IDO. This could assist differentiate between cold and hot cancers and give therapy targets.

In the end, there are still shortcomings in this study. Further *in vivo* and *in vitro* research is needed, which is our subsequent plan, to confirm the potential of necroptosis-related lncRNAs in enhancing the efficacy of immunotherapy in glioma patients. In addition, the expression levels of necroptosis-related lncRNAs in biological specimens have not been conducted, which is also in our future scheme.

## 5 Conclusions

Identifying cold and hot tumors by necroptosis-related lncRNAs can help available immunotherapeutic strategies to achieve efficacy in the precise treatment of individuals. Prior treatment failure can be overcome by targeting necroptosis and associated lncRNAs. Therefore, the mechanisms and relationships among necroptosis, lncRNAs, immunity, and glioma deserve to be fully elucidated and validated.

## Data availability statement

The original contributions presented in the study are included in the article/**Supplementary Material**. Further inquiries can be directed to the corresponding authors.

## Author contributions

Conceptualization, YC and ZN; methodology, KC and FS; software, KC and FS; validation, KC, FS, and XS; formal analysis, KC, FS, and XJ; investigation, KC, and XS; resources, KC and FS; data curation, KC, FS, and XS; writing—original draft preparation, KC, FS; writing—review and editing, ZN and YC; visualization, KC and FS; supervision, YC; project administration, KC and YC; funding acquisition, YC and ZN; All authors contributed to the article and approved the submitted version.
