# Supplementary material for: Necroptosis-related lncRNAs: establishment of a gene module and distinction between the cold and hot tumors in glioma
Source: Front Oncol. 2023 Apr 21;13:1087117. doi: 10.3389/fonc.2023.1087117 (PMC10160458; doi:10.3389/fonc.2023.1087117)
Supplement: Supplementary file 1 [file Table_1.docx]

Supplement Table 1 Real-time quantitative PCR primer sequences used in this study

| **Primer name** | **F primer (5'-3')** | | **R primer (5'-3')** |
| --- | --- | --- | --- |
| β-Actin | GGCCAACCGCGAGAAGATGAC | GGATAGCACAGCCTGGATAGCAAC | |
| CRNDE | ACATGGAAAAATCAAAGTGCTCG | TCTTCTGCGTGACAACTGAGG | |
| FOXD2-AS1 | CCACGCTTCAAAATCCCTGC | AGTGTGGCCTGAGAATGAGC | |
| GNAS-AS1 | AGGTGCGTTACCAGATTGCT | CCATTTTCAGCACGGGTAGAG | |
| LINC01545 | AGAGCAACACTGAACACTTTCC | TCTTGACATCTTCCCACTTTTCC | |
